# Systematic review of clinical effectiveness, components, and delivery of pulmonary rehabilitation in low-resource settings

**DOI:** 10.1038/s41533-020-00210-y

**Published:** 2020-11-19

**Authors:** GM Monsur Habib, Roberto Rabinovich, Kalyani Divgi, Salahuddin Ahmed, Samir Kumar Saha, Sally Singh, Aftab Uddin, Md. Nazim Uzzaman, Hilary Pinnock

**Affiliations:** 1 Bangladesh Primary Care Respiratory Society (BPCRS), Khulna, Bangladesh; 2 NIHR Global Health Research Unit on Respiratory Health (RESPIRE), Usher Institute, University of Edinburgh, Edinburgh, UK; 3 ELEGI/Colt Laboratory, Centre for Inflammation Research, QMRI, The University of Edinburgh and Respiratory Department, Royal Infirmary Edinburgh, Edinburgh, UK; 4 Chest Research Foundation, Pune, India; 5 Johns Hopkins University-Bangladesh, Projahnmo, Dhaka, Bangladesh; 6 Dhaka Shishu Hospital, Dhaka, Bangladesh; 7 Pulmonary and Cardiac Rehabilitation, Department of Respiratory Medicine (Acute Division), University Hospitals of Leicester NHS Trust, Leicester, UK; 8 International Centre for Diarrhoeal Disease Research, Bangladesh (icddr,b), Dhaka, Bangladesh

**Keywords:** Medical research, Rehabilitation

## Abstract

Pulmonary rehabilitation (PR) is a guideline-recommended multifaceted intervention that improves the physical and psychological well-being of people with chronic respiratory diseases (CRDs), though most of the evidence derives from trials in high-resource settings. In low- and middle-income countries, PR services are under-provided. We aimed to review the effectiveness, components and mode of delivery of PR in low-resource settings. Following Cochrane methodology, we systematically searched (1990 to October 2018; pre-publication update March 2020) MEDLINE, EMBASE, CABI, AMED, PUBMED, and CENTRAL for controlled clinical trials of adults with CRD (including but not restricted to chronic obstructive pulmonary disease) comparing PR with usual care in low-resource settings. After duplicate selection, we extracted data on exercise tolerance, health-related quality of life (HRQoL), breathlessness, included components, and mode of delivery. We used Cochrane risk of bias (RoB) to assess study quality and synthesised data narratively. From 8912 hits, we included 13 studies: 11 were at high RoB; 2 at moderate RoB. PR improved functional exercise capacity in 10 studies, HRQoL in 12, and breathlessness in 9 studies. One of the two studies at moderate RoB showed no benefit. All programmes included exercise training; most provided education, chest physiotherapy, and breathing exercises. Low cost services, adapted to the setting, used limited equipment and typically combined outpatient/centre delivery with a home/community-based service. Multicomponent PR programmes can be delivered in low-resource settings, employing a range of modes of delivery. There is a need for a high-quality trial to confirm the positive findings of these high/moderate RoB studies.

## Introduction

The epidemiological transition from communicable to non-communicable disease (NCD) imposes a ‘double burden’ on low- and middle-income countries (LMICs)^1^, which continue to combat infectious diseases but are typically not yet ready to manage NCDs including chronic respiratory diseases (CRDs)^2^. CRDs are common^3,4^ and disabling^5–7^ imposing a substantial burden in LMICs. Poor awareness and insufficient resources^8–10^ in terms of infrastructure for diagnosis, availability of essential drugs, skilled health professionals, and overall healthcare priorities^5^ limit management options^11^.

Pulmonary rehabilitation (PR) is an effective component of CRD care^12^. PR is a comprehensive, multidisciplinary, individually tailored intervention designed to overcome the deconditioning induced by CRDs^13^. The components of PR include, but are not limited to, exercise programmes, chest physiotherapy, education, and supporting self-management and lifestyle change, after optimising the recommended pharmacotherapy^13–15^. PR cost-effectively reduces symptoms, morbidity, hospital admission (and readmission), duration of hospital stay, and emergency medical help and improves functional exercise capacity and health-related quality of life (HRQoL)^16–20^.

However, most of the evidence is generated from high-income countries (HICs) and is disease specific^21–24^ (most commonly chronic obstructive pulmonary disease (COPD)), whereas respiratory disease is often much less differentiated in LMICs. In addition, PR services as developed in HICs may not be deliverable in the same format in LMICs^25,26^ with substantial differences in resources, awareness, culture, healthcare configuration, and profile of diseases^27,28^, which may affect overall management strategy. The potential gains to individuals and healthcare economies, however, are large given the burden of disease in LMICs^29,30^.

Despite well-established effectiveness^19,23^, PR services are often unavailable even in HICs^31–33^ and uptake (by clinicians and patients) is poor particularly in LMICs and especially in rural communities^34^. A strategy is needed to elaborate PR programmes that are deliverable and effective in LMICs. We therefore aimed to systematically search the literature to: (1) assess the impact of PR on HRQoL and exercise capacity, when delivered in low-resource settings for people with CRD, (2) identify the components used in effective interventions, and (3) describe the models of care deliverable in low-resource settings.

## Results

### Study selection

**Fig. 1:**
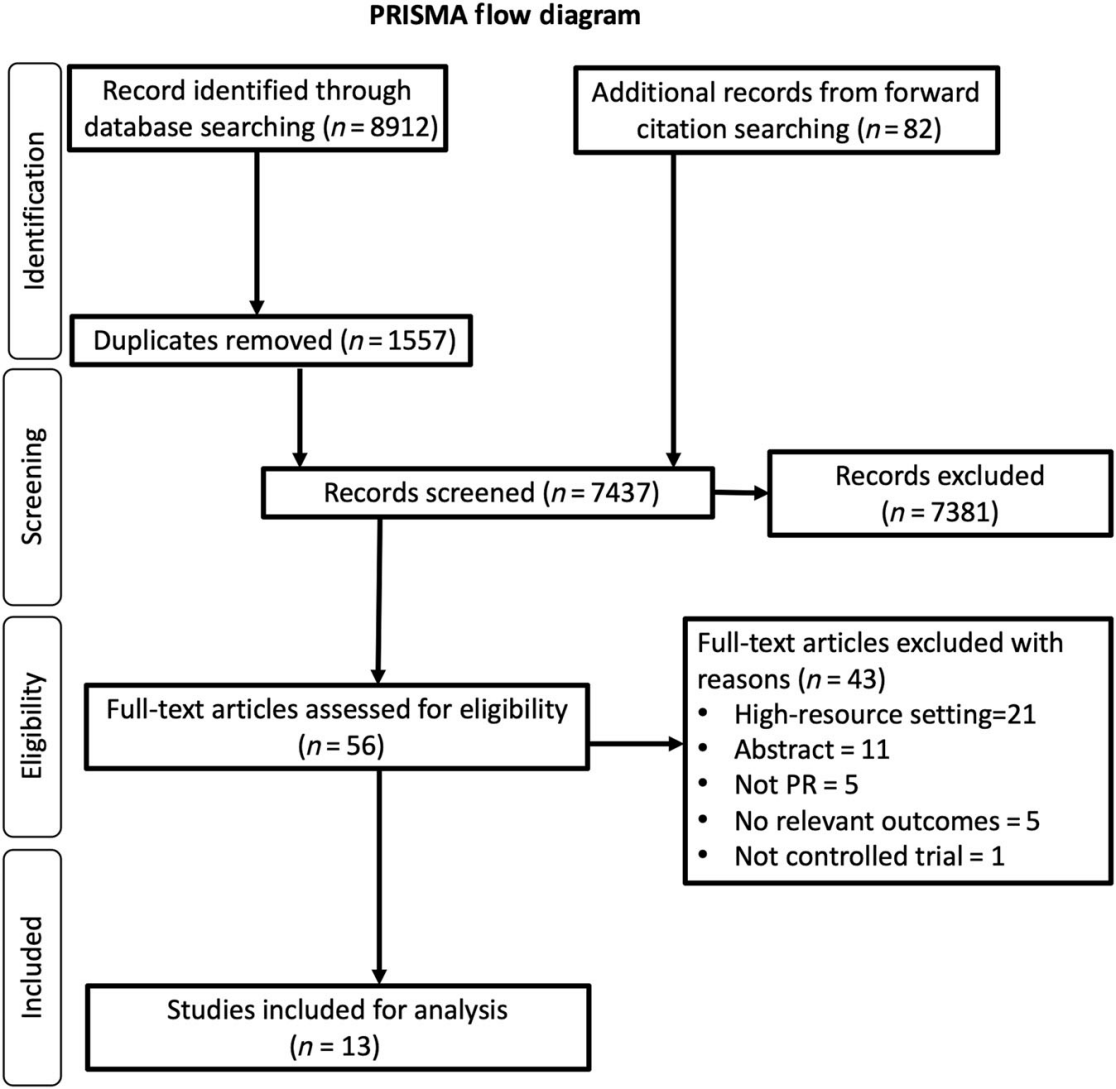
PRISMA flow diagram. Flowchart reporting the number of articles identified, screened, excluded and included.

Our systematic review identified 8912 records. We also found an additional 82 records from forward citation. Following the removal of duplicates, 7437 titles and abstracts were screened (Fig. 1). Fifty-six articles were reviewed in full text, with 43 articles excluded. Thirteen articles met the review criteria and were included^35–47^. No additional papers were identified in the pre-publication update. Total recruitment for the study was 661 individuals with CRD. Attrition was reported in 9 studies; 96 (20%) of the 479 subjects dropped out.

### Study participants

Study participants were COPD patients^35,37–47^ of varying degree of severity in all the trials except one which recruited people with pulmonary impairment after TB (PIAT)^36^. Total number of enrolled participants was 661 of which COPD and PIAT were 83% and 17%, respectively.

### Geographical area

The trials were conducted in Turkey (*n* = 4)^35,39,40,43^, Brazil (*n* = 3)^37,41,46^, India (*n* = 2)^38,47^, Egypt (*n* = 1)^42^, Iran (*n* = 1),^44^ South Africa (*n* = 1)^36^, and Venezuela (*n* = 1)^45^.

### Study settings

Five studies were conducted at hospital outpatient departments^37–39,43,45^ with or without continuation of exercise at home, seven were home-based^35,36,40,42,44,46,47^ training with or without telephonic/face-to-face monitoring or supervision, and one trial was conducted in a community centre^41^. Wherever the PR was delivered, all baseline and follow-up data were collected in a hospital/centre setting.

### Risk of bias (RoB) assessment

Overall RoB is shown in the first column of Table 1 and detailed in Supplementary Results 1. Almost all studies were at overall high RoB, with only two studies^36,39^, which concealed randomisation and took steps to avoid other biases, at moderate RoB. Due to the nature of the intervention, blinding of the patients or the personnel delivering the PR was not possible, but only one study explicitly stated that outcome assessment was blind to allocation^36^. Attrition was a problem or was not clear in all but three studies^39,41,46^. None of the studies had a published protocol, so selective reporting could not be assessed.

**Table 1:** Summary table of included trials with key characteristics, main findings, and interpretation.

Author (year); Country; Intervention; Design; Duration; Risk of bias (RoB)	Chronic respiratory condition; Age: Mean (SD); Inclusion criteria; Recruited/completed	PR baseline assessment	Clinical outcomes FUNCTIONAL EXERCISE CAPACITY HEALTH RELATED QUALITY OF LIFE (HRQoL) BREATHLESSNESS	Comments and conclusion for the harvest plot
de Grass 2014; South Africa; 6w CHC to home PR: exercise + education; RCT: PR vs UC; FU: 6w; MODERATE RoB	Post pulmonary TB; Age: 18–65 years; Ambulant patient contactable by telephone; Recruited: 102 (PR = 51, UC = 51); Analysed: 67 (PR = 33, UC = 34)	Spirometry_;_ 6-MWT; mBorg; EQ-5D; Par-Q	FUNCTIONAL EXERCISE CAPACITY No between-group difference in 6-MWT (m (SD)): Adjusted: NS • PR pre: 401.2 (96.1); post: 411.0 (79.8) • UC pre:340.0 (104.7); post: 356.9 (78.7) HRQoL not assessed BREATHLESSNESS No between-group difference in mBorg mean (SD): Adjusted NS • PR pre: 10.1 (2.3); post: 10.4 (1.8) • UC pre: 11.4 (1.6): post: 11.24 (1.5)	Significant difference lost when adjusted for large baseline differences. Attrition 35%: similar in both groups FUNCTIONAL EXERCISE CAPACITY^a^ Illustrated as no significant changes (no effect) BREATHLESSNESS^a^ Illustrated as no significant changes (no effect)
Duruturk 2015; Turkey; 6w Hospital OPD PR: cycle ergometry training or callisthenic exercises; Three groups of RCT: PR^Cycle^ vs PR^Cali^ vs UC FU: 6w; MODERATE RoB	Mod/Severe COPD; Age: PR^Cycle^ = 61 years, PR^Cali^ = 61 years, vs UC = 64 years No cCI to PR Recruited: 47 (PR^Cycle^ = 16, vs PR^Cali^ = 16, vs UC = 15); Analysed: 42 (PR^Cycle^ = 15, vs PR^Cali^ = 14, vs UC = 13)	Spirometry; Cycle ergometry; FT; ECG; mMRC	FUNCTIONAL EXERCISE CAPACITY PR^Cycle^ and PR^Cali^ Between-group difference^b^ in 6-MWT (mean (SD)): *p* < 0.001 • PR^Cycle^pre: 448.7 (60.9); post: 514.2 (59.3) • PR^Cali^pre: 395.6 (98.2); post: 482.3 (65.4) • UC pre: 413.6 (125.8); post: 413.5 (121.8) HRQoL PR^Cycle^and PR^Cali^ Between-group difference in SGRQ (mean (SD)): *p* = 0.001 • PR^Cycle^ pre: 49.3 (19.6); post: 28.7 (12.9) • PR^Cali^ pre: 49.3 (19.6); post: 26.7 (15.9) • UC pre: 45.6 (15.0); post: 45.4 (13.7) BREATHLESSNESS PR^Cycle^and PR^Cali^ Between-group difference in mMRC (mean (SD)): *p* < 0.001 • PR^Cycle^ pre: 3.3 (0.9); post: 1.8 (0.6) • PR^Cali^ pre: 2.9 (1.0); post: 1.8 (0.8) • UC pre: 2.6 (0.8); post: 2.7 (0.8)	Three groups, small numbers but minimal attrition FUNCTIONAL EXERCISE CAPACITY^a^ Illustrated as a significant positive effect HRQoL^a^ Illustrated as a significant positive effect BREATHLESSNESS^a^ Illustrated as a significant positive effect
Deepak 2014; India; 12w Hospital OPD: exercise + education; RCT: PR vs UC; FU: 12w; HIGH RoB	Males recruited 2w post AECOPD; Age: PR = 58.4 (6.8); UC = 59.4 (6.7); Recruited: 60 (PR = 30, UC = 30); Analysed: 56 (PR = 28, UC = 28)	Spirometry; 6-MWT; mMRC; SGRQ; ABG	FUNCTIONAL EXERCISE CAPACITY Within-group change in 6-MWT (m (SD)) • PR pre: 303.1 (84.5); post: 340.5 (86.2); *p* < 0.001 (improved) • UC pre: 288.3 (96.1); post: 260.0 (100.2); *p* < 0.001 (worsened) HRQoL Within-group change in SGRQ (mean (SD)) • PR pre: 53.7 (12.9); post: 39.0 (12.9); *p* < 0.001 (improved) • UC pre: 57.3 (18.5); post: 62.6 (18.7); *p* < 0.002 (worsened) BREATHLESSNESS Within-group change in mMRC • PR improved; *p* < 0.013 • UC not improved; *p* < 0.102	Minimal attrition. Between-group significance not reported FUNCTIONAL EXERCISE CAPACITY^b^ Illustrated as a significant improvement in PR group (worsened in UC group) HRQoL^b^ Illustrated as a significant improvement in PR group (worsened in UC group) BREATHLESSNESS^b^ Illustrated as a significant improvement in PR group (not in UC)
Elci 2008; Turkey; 12w Hospital OPD (+home): exercise + education; RCT: PR vs UC; FU: 4, 8, 12w; HIGH RoB	Patient with GOLD -defined COPD Age: PR = 59.7 (8.6); UC = 58.1 (11.5); Recruited: 78 (PR = 39; UC = 39); Analysed: NR	Spirometry; 6-MWT; SGRQ; mMRC; HADS; SF-36	FUNCTIONAL EXERCISE CAPACITY Within-group change in 6-MWT (m (SD)) • PR pre: 312.4 (56.3); post: 328.9 (48.8); *p* = 0.001 (improved) • UC pre: 305.1 (54.6); post: 298.2 (52.8); *p* = 0.001 (worsened) HRQoL Between-group difference in SGRQ (mean (SD)); *p* = 0.001 • PR pre: 60.3 (18.2); post: 45.9 (11.6) • UC pre: 61.7 (19.9); post: 65.5 (17.4) BREATHLESSNESS Within-group change in mMRC (mean (SD)); PR pre: 3.2 (0.6); Post: 2.89 (0.7); *p* = 0.001 (improved) UC: not reported	Attrition not reported. Between-group significance for 6-MWT and mMRC not reported FUNCTIONAL EXERCISE CAPACITY^b^ Illustrated as a significant improvement in PR group (worsened in UC) HRQoL^a^ Illustrated as a significant positive effect BREATHLESSNESS^b^ Insufficient information to estimate the change as the data of UC is not reported
Akinci 2011; Turkey; 12w Home + tel. support: exercise + education; CCT: PR vs UC; FU: 12w; HIGH RoB	Clinically stable, severe/very severe COPD; Age: PR = 71.8 (7.8); UC = 65.1 (10.2); Recruited: 52 (PR = 27; UC = 25); Analysed: 32 (PR = 16; UC = 16)	Spirometry 6-MWT SGRQ, BDI; ABG	FUNCTIONAL EXERCISE CAPACITY Within-group change in 6-MWT (m (SD)): • PR pre: 157.9 (64.5); post: 190.3 (65.0); *p* = 0.001 (improved) • UC pre: 176.3 (54.9); post: 170.6 (55.4); *p* = 0.16 (NS) HRQoL Within-group change in SGRQ (mean (SD)) • PR pre: 55 (16); post: 37 (13); *p* = 0.001 (improved) • UC pre: 45 (18); post: 47 (16); *p* = 0.06 (NS) BREATHLESSNESS Within-group change in BDI (mean (SD)) PR pre: 5.2 (1.6); post: 7.9 (1.5); *p* = 0.001 UC pre: 6.1 (2.1); post: 5.9 (1.5); *p* = 0.35	Intervention group worse at baseline. Attrition is approximately 40% in both groups. Between-group significance not reported FUNCTIONAL EXERCISE CAPACITY^b^ Illustrated as a significant improvement in PR group (no significant change in UC) HRQoL^b^ Illustrated as a significant improvement in PR group (no significant change in UC) BREATHLESSNESS^b^ Illustrated as a significant improvement in PR group (no significant change in UC)
Farias 2014; Brazil; 8w Local park: exercise + education (hospital); RCT: PR vs UC; FU: 8w; HIGH RoB	COPD patients Age: PR = 64.6 (10.1); UC = 70.5 (8.1); Recruited: 38 (PR—19; UC—19); Analysed: 34 (PR—16; UC—18)	Spirometry 6-MWT; SGRQ; BODE index	FUNCTIONAL EXERCISE CAPACITY Within-group change in 6-MWT (m (SD)) • PR pre: 430.0 (80.6); post: 472.0 (72.7) *p* < 0.05 (improved) • UC pre: 383 (72.5); post: 331.8 (86.7) *p* = NS HRQoL Within-group change in SGRQ (mean (SD)) • PR pre: 42.8 (SD 14.7); post: 26.4 (SD 7.3) *p* < 0.05 • UC pre: 55 (17); post: 64.3 (12) *p* = NS Text states ‘significantly different intergroup scores after the intervention—but no data BREATHLESSNESS Within-group change in MRC (mean (SD)) • PR pre: 2.3 (0.8); post: 2.0 (0.6) (*p* < 0.05) (improved) • UC pre: 2.8 (0.9); post: 3.3 (08) NS	PR group was younger, less symptomatic, better baseline 6-MWT. Minimal attrition FUNCTIONAL EXERCISE CAPACITY^b^ Illustrated as a significant improvement in PR group (UC worsened—significance NR) HRQoL^b^ Illustrated as a significant positive effect BREATHLESSNESS^b^ Illustrated as a significant improvement in PR group (no significant change in UC)
Paz-Diaz 2007; Venezuela; 8w Hospital OPD; PR: exercise + education; RCT: PR vs UC; FU: 8w; HIGH RoB	Stable, severe COPD; Age: PR = 67 (5); UC = 62(7); Recruited: 24 (PR—10; UC—14) Analysed: NR	Spirometry SGRQ; MRC; Beck Depression Inventory	FUNCTIONAL EXERCISE CAPACITY not assessed HRQoL Within-group change in SGRQ (mean (SD)) • PR pre: 58 (13); post: 45 (12); *p* < 0.001 • UC pre: 55 (16); post: 58 (16); *p* = NS BREATHLESSNESS Within-group change in MRC (mean (SD)) • PR pre: 2.1 (0.5); post: 1 (0.5); *p* < 0.01 • UC pre: 2.1 (0.6); post: 2.1 (0.5); *p* = NS	Attrition is not reported. Between-group significance not reported HRQoL^b^ Illustrated as a significant improvement in PR group (no significant change in UC) BREATHLESSNESS^b^ Illustrated as a significant improvement in PR group (no significant change in UC)
Pradella 2015; Brazil; 8w (1-w hospital then home) PR: exercise + education; RCT: PR vs UC; FU 8w; HIGH RoB	GOLD defined COPD; Age: PR = 62.4 (10.7); UC = 65.3 (8); Recruited: 50 (PR = 32; UC = 18); Analysed: 44 (PR = 29; UC = 15)	Spirometry; 6-MWT; SGRQ	FUNCTIONAL EXERCISE CAPACITY Between-group difference in 6-MWT (m (SD)); MD 60.2 (95%CI 4.6 to 115.7); *p* < 0.05 • PR pre: 485.1 (79.6); post: 550.8 (100.7) • CG pre: 456.5 (71.1); post: 462.1 (101.4) Between-group difference in ESWT: MD 285.42 (7.1 to 563.8) • PR pre: 708.4 (364.4); post: 1025.0 (706.2) • UC pre: 923.7 (588.8); post: 954.9 (572.4) HRQoL Between-group difference in SGRQ (mean (SD)); MD 9.7 (−1.0 to −0.1); *p* < 0.05 • PR pre: 50.3 (20.9); post: 43.6 (18.5) • UC pre: 49.1 (23.2); post: 52.3 (24.5) BREATHLESSNESS Between-group difference in Borg scale (mean (SD)) NS PR pre: 0.24 (0.6); post: 0.13 (0.4) UC pre: 0.26 (0.8); post: 0.33 (0.7)	Rehabilitation group had worse lung function FUNCTIONAL EXERCISE CAPACITY^a^ Illustrated as consistently a significant positive effect HRQoL^a^ Illustrated as a significant positive effect BREATHLESSNESS^a^ Illustrated as no significant changes (no effect)
Karapolat 2007; Turkey; 8w Hospital OPD PR: exercise + education; RCT: PR vs UC; FU: 8w; HIGH RoB	Stable mild/moderate COPD; Age: PR = 65.1 (9.4); UC = 66.6 (8.4); Recruited: 49 (PR = 27; UC = 22); Analysed: 45 (PR = 26; UC = 19)	Spirometry; 6-MWT; SGRQ; ABG; VAS (Dyspnoea)	FUNCTIONAL EXERCISE CAPACITY Within-group change in 6-MWT (m (SD)) • PR pre: 261.6 (41.5); post: 383.2 (50.4); *p* < 0.05 (improved) • UC pre: 226.8 (62.7); post: 241.9 (57.4); NS HRQoL Within-group change in SGRQ (mean (SD)) • PR pre: 45.1 (17.8); post: 28.3 (15.2); *p* < 0.05 (improved) • UC pre: 50.7 (15.7); post: 47.0 (17.3); NS BREATHLESSNESS Within-group change on VAS (mm (SD)) PR pre: 5.9 (2.0); post: 3.1 (1.6); *p* < 0.05) UC pre: 5.3 (2.0); post: 5.8 (1.8); *p* = NS	Five ‘ineligible’ UC participants were excluded after randomisation FUNCTIONAL EXERCISE CAPACITY^b^ Illustrated as a significant improvement in PR group (no significant change in UC) HRQoL^b^ Illustrated as a significant improvement in PR group (no significant change in UC) BREATHLESSNESS^b^ Illustrated as a significant improvement in PR group (no significant change in UC)
De Souto Araujo 2012; Brazil; 8w Hospital physio centre PR: exercise (floor or aquatic based) + education; Three groups of RCT: PR^Fl^ vs PR^Aq^ vs UC; FU: 8w; HIGH RoB	Stable mod/severe/very severe COPD; Clinically stable; Age: PR^Fl^ = 56.9 (7.9); PR^Aq^ = 62.4 (9.9); UC = 71.1 (10.1); Recruited: 42 (PR^Fl^ = 14; PR^Aq^ = 14; UC = 14); Analysed: 32 (PR^Fl^ = 13; PR^Aq^ = 8; UC = 11)	Spirometry; 6-MWT; SGRQ; BODE index; Borg Fatigue score	FUNCTIONAL EXERCISE CAPACITY Within-group change in 6-MWT (m (SD)): • PR^Fl^ pre: 446.5 (114.5); post: 468.8 (106.8); NS • PR^Aq^ pre: 434.6 (121.0); post: 490.9 (137.8); *p* = 0.02 (improved) • UC pre: 393.3 (135.1); post:360.7 (129.4); *p* = 0.02 (worsened) HRQoL Within-group change in SGRQ (data NR) • PR^Fl^ *p* = 0.001 • PR^Aq^ *p* = NS • UC *p* = NS BREATHLESSNESS Within-group change in MRC (data NR) • PR^Fl^ *p* = NS • PR^Aq^ *p* < 0.001 (improved) • UC *p* < 0.05 (worsened)	Differential attrition between the groups. Control group was older. Inter-group comparison all NS, but no paired comparisons (PR^Fl^/UC or PR^Aq^/UC). Some data only illustrated graphically FUNCTIONAL EXERCISE CAPACITY^b^ Illustrated as a significant improvement in PR^Fl^ group, not in PR^Aq^ (no significant change in UC) HRQoL^b^ Illustrated as a significant improvement in PR^Fl^ group, not in PR^Aq^ and UC BREATHLESSNESS^b^ Illustrated as a significant improvement in PR^Aq^ group, not in PR^FG^ (no significant change in UC)
Ghanem 2010; Egypt; 8w Home + hospital 2 weekly PR: exercise + education; RCT: PR vs UC; FU: 8w; HIGH RoB	Mod/severe COPD post admission Age: PR = 56.9 (11.5); UC = 56.43 (9.03); Recruited: 39 (PR = 25; UC = 14); Analysed: 39 (PR = 25; UC = 14)	Spirometry; 6-MWT; CRQ-SAS	FUNCTIONAL EXERCISE CAPACITY Significant between-group difference in 6-MWT (m (SD)): MD 58.2 ± 11.2 (*p* < 0.001) • PR pre: 88.7 (19.1); post: 141.7 (23.1) • UC pre: 83.8 (15.9); post: 68.6 (32.1) HRQoL Between-group significant difference in all CRQ domains (mean (SD)) Fatigue MD 5.3 (1.9–9.8); *p* = 0.004 • PR pre: 9.8 (2.8); post: 17.4 (5.4) • UC pre: 11.6 (6.1); post: 13.2 (5.1) Emotion MD 8.7 (2.5–15); *p* = 0.008 • PR pre: 22.1 (5.8); post: 33.5 (7.2) • UC pre: 27.0 (12.6); post: 29.7 (11.4) BREATHLESSNESS MD 5.5 (3.0–9.0); *p* = 0.003 • PR pre: 11.8 (5.0); post: 19.6 (5.2) • UC pre: 12.4 (4.4); post: 13.5 (4.3)	Unclear why uneven numbers in the groups FUNCTIONAL EXERCISE CAPACITY^a^ Illustrated as a significant positive effect HRQoL^a^ Illustrated as a significant positive effect BREATHLESSNESS^a^ Illustrated as a significant positive effect
Mohammadi 2013; Iran; 8w (1-w in hospital, pre-discharged, then home) PR: exercise + education; RCT: PR vs UC; FU: 8w; HIGH RoB	Mod/severe COPD; Age: NR (though stated to be similar between groups; Recruited: 40 (PR = 20; UC = 20); Analysed: NR	ADL level; SF-12 QOL; FSS	FUNCTIONAL EXERCISE CAPACITY Not measured HRQoL Significant between-group difference in SF-12 (mean (SD)); *p* < 0.001 • PR pre: −21.3 (11.5) post: −14.5 (7.1); • UC pre: −24.6 (9.2); post: −27.1 (8.5); BREATHLESSNESS Not measured	Sample size calculation: 20/group, and 20/group were analysed. No data on number recruited/ attrition HRQoL^a^ Illustrated as a significant positive effect
Singh 2003; India; 4w Hospital then home PR: exercise + education; RCT: PR vs UC; FU: 4w; HIGH RoB	Stable, severe COPD; Age: 59.3 (6.4); Recruited: 40 (PR = 20; UC = 20); Analysed: NR	Spirometry; 6-MWT; CRQ	FUNCTIONAL EXERCISE CAPACITY Within-group change in 6-MWT (m (SD)) • PR pre: 261 (113); post: 315 (118); *p* < 0.001 (improvement) • UC pre: 257.7 (158); post: 264 (157); NS HRQoL a group difference in CRQ (mean (SD)); *p* < 0.001 • PR pre: 2.9 (0.9); post: 3.8 (0.9) *p* < 0.001 • UC pre: 3.1(0.8); post: 3.2 (0.8) NS BREATHLESSNESS Within-group change in dyspnoea domain of CRQ (mean (SD)) • PR pre: 3.16 (1.0); post: 4.1 (0.9); *p* < 0.001 • UC pre: 3.5 (0.8; post: 3.6 (0.8)	Baseline characteristics not given but reported as not significantly different. Attrition not reported FUNCTIONAL EXERCISE CAPACITY^b^ Illustrated as a significant improvement in PR group (no significant change in UC) HRQoL^b^ Illustrated as a significant improvement in PR group (no significant change in UC) BREATHLESSNESS^b^ Illustrated as a significant improvement in PR group (no significant change in UC)

^a^Solid in the harvest plot to show between-group comparison.

^b^Hatched in the harvest plot to show within-group comparison.

*CCT* controlled clinical trial, *CG* control group, FG floor group, *6-MWT* 6-min walk test, *EQ-5D* EuroQual Questionnaire, *Par-Q* Physical Activity Readiness Questionnaire, *RoB* risk of bias, *HRQoL* health-related quality of life, *SGRQ* Saint George Respiratory Questionnaire, *cycle ergo* cycle ergometry, *FT* Fitness Test, *SF-36* Short Form-36, *HADS* Hospital Anxiety and Depression Scale, *BDI* Baseline Dyspnoea Index, *ABG* arterial blood gas, *m* metres, *MD* mean difference, *VAS* Visual Analogue Scale, NS not significant.

### Effectiveness of intervention (Objective 1)

Although 6-min walking test (6-MWT), St George’s Respiratory Questionnaire (SGRQ), and modified Medical Research Council (mMRC) were widely used to assess functional exercise capacity, HRQoL, and breathlessness respectively, only six of the trials presented between-group comparisons^36,39,40,42,44,46^. The other seven provided within-group differences^35,37,38,41,43,45,47^. In addition, heterogeneity in terms of mode of intervention, duration, setting, comparator, and baseline measurements confirmed our decision that meta-analysis was not appropriate.

**Fig. 2:**
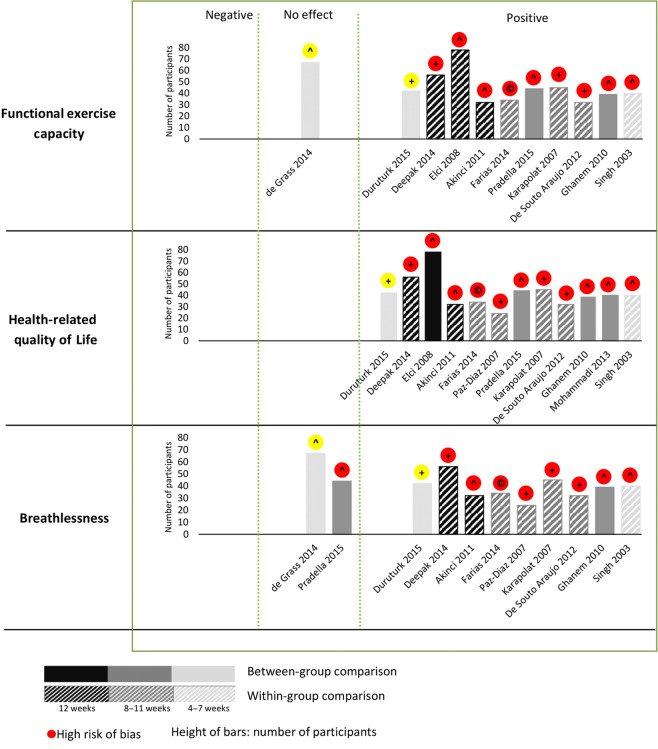
Harvest plot illustrating the impact of pulmonary rehabilitation on functional exercise capacity, health-related quality of life, and breathlessness. Each column represents an included study, shaded according to whether it is a RCT (solid shading) or within group comparison (hatched shading). The depth of shading represents study duration of 4-7 weeks (light shading); 8-11 weeks (moderate shading); 12 weeks or more (dark shading). The height of the bars represent the number of patients. The icon on the top of the bars represents the overall risk of bias as high risk of bias (red) or moderate risk of bias (yellow). Within the icon the mode of delivery of the PR is indicated as + (OPD-based); ^ (Home-based) or C (Community-based). The effectiveness of interventions is illustrated with respect to functional exercise capacity, health-related quality of life, and breathlessness in the three tiers of the graph. Studies are positioned according to whether overall the outcomes were positive (i.e., interventions were significantly beneficial), negative (i.e., interventions were significantly harmful), or had no effect. Table 1; Column 5 details how these decisions were reached.

We therefore undertook a narrative synthesis and illustrated functional exercise capacity, HRQoL, and breathless in a harvest plot (Fig. 2). Our interpretation of the study findings and the structured process determining the decisions that underpinned the harvest plot are described in column 5 of Table 1.

Changes in functional exercise capacity were measured in 11 studies^35–43,46,47^. Significant positive changes were found in 10 studies^35,37–43,46,47^; the exception being one of the two studies at moderate RoB^53^. HRQoL was measured in 12 studies^35,37–47^; all showing positive changes. Breathlessness was measured in 11 studies^35–39,41–43,45–47^ of which 9 studies^35,37–39,41–43,45,47^ showed significant positive changes and 2 studies (1 at moderate RoB)^36,46^ showed no changes after intervention. None of the studies reported negative effects after the intervention.

### Components of the intervention (Objective 2)

All interventions included exercise and non-exercise components (as per inclusion criteria), though the approach, content, method of delivery, and duration varied. The components are described in Table 1 and their presence are indicated in a matrix in Table 2.

**Table 2:** Components of pulmonary rehabilitation from the selected papers.

	de Grass 2014	Duruturk 2015	Deepak 2014	Elci 2008	Akinci 2013	Farias 2014	Paz-Diaz 2007	Pradella 2015	Karapolat 2007	de Souto Araujo 2012	Ghanem 2009	Mohannadi 2013	Singh 2003	
Exercise programme														
Endurance training (including interval training)	✓	✓	✓	✓	✓	✓	✓	✓	✓	✓	✓	✓	✓	13
Resistance/strength training		✓	✓	✓				✓	✓	✓	✓			7
Upper limb exercise	✓	✓			✓		✓	✓		✓				6
Flexibility training		✓					✓		✓		✓			4
Breathing exercises (including IMT)	✓		✓		✓		✓		✓		✓	✓	✓	8
Other components														
Pursed-lip breathing	✓		✓		✓		✓	✓	✓		✓	✓	✓	9
Diaphragmatic breathing	✓		✓		✓		✓	✓			✓		✓	7
Knowledge (disease/medication)	✓	✓	✓	✓	✓	✓		✓	✓		✓	✓		10
Skill acquisition (airway clearance, inhaler technique, use of oxygen)	✓	✓		✓	✓				✓		✓		✓	7
Psychological interventions (CBT, relaxation)				✓	✓			✓	✓					4
Coping strategies (pacing, energy conservation)			✓	✓	✓				✓				✓	5
Nutrition				✓					✓		✓	✓		4
Physical activity (Unsupervised exercise)								✓	✓			✓		3
Smoking cessation					✓							✓		2
Self-management											✓			1
Social support (including walking aids)			✓											1
Pharmacological optimisation					✓					✓				2

Endurance training was included in all 13 studies. Other common exercises were upper limb exercise^35–37,39,45,46^ and strength training in seven studies^37–40,42,43,46^ and stretching exercises in four studies^39,42,43,45^. Although not described in detail, the other common component was breathing exercises included in eight studies^35,36,38,42–45,47^. Along with the exercise, patient education was provided in ten studies^35,36,38–44,46^, and skills (such as inhaler technique and airway clearance) were included in seven studies^35,36,39,40,42,43,47^. Other components in a minority of studies were social support^38^, optimisation of pharmacotherapy^35,37^, nutrition^40,42–44^, coping strategies^35,38,40,43,47^, psychological intervention^35,40,43,46^, self-management^42^, and physical activity interventions^43,44,46^. Smoking cessation support was reported in only two studies^35,44^.

### Models of care (Objective 3)

We identified three models of PR service in our included studies according to the settings in which they were delivered (see Table 3). Five were based in hospital or rehabilitation centres^37–39,43,45^, and one was based in a community health centre^41^. Only one was delivered completely at home^35^ while most home-based programmes^36,40,42,44,46,47^ provided initial training in the hospital or centre and maintained telephone^40,44,46^ or face-to-face supervision^42,47^. The programmes typically lasted 8 weeks (range 4–12), with supervised sessions lasting between 30 and 120 min provided 2 or 3 times per week. Home-based programmes promoted more frequent exercise sessions often supported by telephone or face-to-face contacts. Physiotherapists provided the sessions in six studies^36,38–41,43^, with nurses involved in four studies^35,40,42,44^. Adherence to the PR course was poorly reported with no details provided about reasons for non-completion.

**Table 3:** Models of pulmonary rehabilitation services.

Study	Who	Where	Whom	How	What (components of PR are described in Table 2)
de Grass 2014	Assessor: physiotherapist Provider: physiotherapist	Assessment (0, 3, 6 weeks): community health centre PR: initial training: community health centre, then home	Post-TB patients after active treatment	• PR course: 6 weeks • Frequency: Daily • Duration of sessions: NR	Home exercise, physiotherapy/breathing exercises, education materials
Duruturk 2015	Assessor: physiotherapist Provider: physiotherapist	Assessment (0, 6 weeks): hospital PR: hospital	Moderate/severe/stable COPD patients	• PR course: 6 weeks • Frequency: 3 times a week • Duration of sessions: 30 min	Exercise training Physiotherapy/breathing exercises, Education session
Deepak 2014	Assessor: NR Providers: physiotherapist, doctor	Assessment (0, 12 weeks): hospital PR: hospital	COPD patients 2 weeks after hospital discharge	• PR course: 12 weeks • Frequency: NR • Duration of session: 2 h	Exercise training Physiotherapy/breathing exercises Education sessions Psycho-social support
Elci 2008	Assessor: nurse Providers: physiotherapist, doctor	Assessment (0, 4, 8, 12 weeks): hospital PRP: hospital (+ home exercises)	Stable COPD patients	• PR course: 12 weeks • Frequency: 2 times a week • Duration of sessions: 90 min	Exercise training Physiotherapy/breathing exercises Education sessions + materials
Akinci 2011	Assessor: doctor Provider: nurse trained in PR	Assessment (0, 12 weeks): hospital PR: home + telephone support	COPD patients	• PR course: 12 weeks • Frequency: Daily exercise • Duration of home visits: 90 min	Exercise training + home exercise Physiotherapy/breathing exercises education sessions
Farias 2014	Assessor: physiotherapist Provider: physiotherapist	Assessment: (0, 8 weeks): hospital PR: supervised in local park (education at hospital)	COPD patients	• PR course: 8 weeks • Frequency: Five times a week • Duration of exercise sessions: 40–60 min	Exercise: walking in local park Physiotherapy/breathing exercises Education sessions
Paz-Diaz 2007	Assessor: NR Provider: NR	Assessment (0, 8 weeks): hospital PR: hospital	Stable, severe COPD	• PR course: 8 weeks • Frequency: 3 times per week • Duration of PR: 60 min	Exercise training Physiotherapy/breathing exercises
Pradella 2015	Assessor: NR Provider: NR	Assessment: (0, 8 weeks): rehabilitation centre PR: 1-week rehabilitation centre, then home + telephone support	COPD patients	• PR course: 8 weeks • Frequency: 3 times a week • Duration of sessions: 90 min	Exercise (walking and stairs) Physiotherapy/breathing exercises Printed material
Karapolat 2007	Assessor: doctor Provider: physiotherapist	Assessment (0, 8, 12 weeks): hospital PR: hospital	Mild, moderate, and severe stable COPD	• PR course: 8 weeks • Frequency: 3 times a week • Duration of sessions: 90 min	Exercise, Physiotherapy/breathing exercises Education
De Souto Araujo 2012	Assessor: NR Provider: NR	Assessment: (0, 8 weeks) physiotherapy centre PR: physiotherapy centre	Moderate, severe, and very severe stable COPD	• PR course: 8 weeks • Frequency: 3 times a week • Duration of PR sessions: 90 min	Exercise (floor or pool) Optimisation of pharmacotherapy
Ghanem 2010	Assessor: doctor, nurses Provider: pulmonary specialist, nurses	Assessment: (0, 8 weeks); in hospital pre-discharge PR: home + hospital 2 weekly	Post-exacerbation COPD patients	• PR course: 8 weeks • Frequency: Every other day • Duration of sessions: NR	Exercise, Physiotherapy/breathing exercises Education
Mohammadi 2013	Assessor: nurse specialist Provider: nurse at home	Assessment: (0, 8 weeks) PR: 1-week in hospital pre-discharge then home + telephone alternate days	Post-exacerbation COPD patients	• PR course: 8 weeks • Frequency: Alternate days; • Duration of PR sessions: NR	Exercise, Physiotherapy/breathing exercises Education (3 1-h sessions)
Singh 2003	Assessor: NR Provider: NR	Assessment: (0, 4 weeks) hospital PR: hospital then home + weekly supervision	Stable, severe COPD	• PR course: 4 weeks • Frequency: Twice a day • Duration of PR sessions: 30 min	Exercise, Physiotherapy/breathing exercises

Inexpensive instruments were often used in the studies, which ensured the wide availability and acceptability to the consumers. Lower limb endurance exercise was conducted by walking as opposed to expensive stationary bicycle with upper limb resistance/strength training conducted using home-made weights, such as water bottles. Breathing exercises were done with similar devices that are used in higher resource setting (e.g. incentive spirometers, tri-flow).

## Discussion

In summary, our systematic review identified and selected 13 heterogeneous studies from 7 different countries with a total study population of 661 patients. Overall, PR was reported as being effective in terms of improving functional exercise capacity, HRQoL, and breathlessness, though RoB was high in 11 studies. Of the two at moderate RoB, one showed no benefit in any of the outcomes reported^36^. The exercise programmes typically included endurance, interval, upper limb, and resistance/strength training. The commonest additional components were education to improve knowledge and skill acquisition (e.g. inhaler technique) and strategies for coping with breathlessness. Smoking cessation was provided in only two studies. Most PR services were provided in hospital settings or home based, with some describing adaptations to locally acceptable and deliverable approaches.

The strength of this systematic review is its broad literature search constructed with the help of a senior librarian and informed by Cochrane’s standard search terms for COPD and LMICs. Nevertheless, we may have missed important studies of PR conducted in low-resource settings. Although we did not specifically search for papers in other languages, we were open to including non-English language papers but none were identified in our searches, perhaps because locally conducted studies or articles in local languages are often not published in indexed journals^48^. We may have missed important information from these studies but lacked resources to extend the search to non-indexed publications and grey literature.

We followed rigorous Cochrane methodology duplicating the selection, data extraction, and quality assessment procedures, but confidence in our findings is limited by the high RoB in most of the studies included. We only included controlled trials because we wanted to assess effectiveness. We acknowledge, however, that in LMICs there are many challenges and barriers such as lack of infrastructure, heterogeneity of resources, and poor health literacy, which discourage clinical trials^49,50^. Reliable tools for measuring outcomes (e.g. validated questionnaires in local language, well-trained assessors, effective training facilities, etc.) may not be available in low-resource settings reducing accuracy of assessing effectiveness^51,52^. We did not search for health economic assessments.

All our included studies reported positive outcomes, but the high RoB limits interpretation of this finding. In contrast, the evidence from studies conducted in HICs are mostly at low-to-moderate RoB, so that the Cochrane review was able to conclude confidently that PR was an effective intervention for people with COPD^23^. It is likely that insufficient resources, training, and facilities in LMICs is responsible for the lack of high-quality trials. This is a gap that NIHR-funded initiatives, such as RESPIRE^53^, and RECHARGE^54^ aim to address.

Compared to high-resource settings, under-diagnosis due to lack of awareness of CRD compounded by limited access to diagnostic tools such as spirometry results in a minority of potentially eligible participants being approached to be enrolled in studies. Poor universal health coverage^55^ and ‘catastrophic’ costs of healthcare^56^ further limit participation in trials.

The lack of diagnostics means that patients recruited as COPD may in fact have a range of undifferentiated CRDs (e.g. pulmonary impairment after tuberculosis or combined obstructive and restrictive disorder^57^). While this lack of detailed characterisation may impact on findings, offering PR to people with CRD (regardless of specific diagnosis) may be a more appropriate strategy especially in resource-limited settings.

There was considerable variation in the clinical status of participants, which might affect outcomes. There was considerable range in severity of functional limitation (see Table 1). In addition, some of the patients were stable at enrolment^37,39,40,43,45,47^ while some had been hospitalised for a recent exacerbation^38,42,44^.

Exercise training is the cornerstone of PR^58^ and was an inclusion criterion for the studies in our review. Endurance training was included in all the studies in addition to a range of other modalities as per recognised guidelines. Behavioural changes and continuing physical activities are crucial for maintaining effectiveness of PR^59^, but these were not reported in any of the studies.

Education on CRD and its treatment was widely provided along with strategies on managing breathlessness, but other components such as self-management support and addressing social care needs were rarely reported, despite evidence of effectiveness in CRDs^60^. In HICs, smoking is the predominant risk factor and cessation support is seen as essential. Surprisingly, only two of the studies in our review reported a smoking cessation component and none reported avoidance of pollution and indoor biomass exposure, which are also important risk factors in LMICs^61,62^. The brief descriptions in the papers make it difficult to assess how these and other important educational topics (such as inhaler technique) were addressed.

Models of PR delivery depends on who, where, to whom, and how the service is delivered^63^. Different models of PR services were described in the included studies reflecting diversity in the healthcare context and access to PR services; individuals’ health literacy; and background beliefs, attitudes, and preferences, as well as practical factors such as availability of transport and capability of payment^64^. A home-based, inexpensively equipped PR service with minimal attendance at a potentially distant centre may be more suitable model in rural areas with limited resources and poor transport infrastructure^65,66^. In home-based models, the cost to the patient is minimised, and people have flexibility in how they invest their time^67–69^. Digital technology is a rising paradigm in LMICs, which may be considered in developing a remote model of PR service^70^.

Our findings have implications for clinical practice and research. Breathlessness is the principal symptom that drives the patients with CRDs to seek medical help^71^. In LMICs, diagnosis of chronic respiratory symptoms depends on clinical history and physical examination, with limited, or sometimes no, access to spirometry or other investigations^72^. Poor healthcare coverage may mean that tasks regarded as prerequisites to referral in HICs, such as identifying co-morbidities, optimising pharmacotherapy, and exclusion of contraindications, may need to be a component of PR in LMICs^73^. The studies included in this review identified some practical solutions to these challenges, but high-quality evidence of the clinical and cost effectiveness of these pragmatic approaches is urgently needed.

In conclusion, recommendations in PR guidelines typically reflect services delivered in high-income settings. Our literature review, although identifying studies with high-to-moderate RoB, highlighted the feasibility of conducting PR in LMICs with positive effects on outcomes such as exercise tolerance, HRQoL, and symptoms improvement. Our findings point to the need for PR services that are effective across a broad range of (potentially poorly differentiated) CRDs, overcoming barriers of cost, distance, and access to healthcare such that they are deliverable and sustainable in low-resource settings with minimal equipment. Only then will the known benefits of PR be available to address the increasing burden of CRDs in LMICs.

## Methods

### Published review protocol

The review is registered with PROSPERO [ID: CRD42019125326]. The detailed systematic review protocol is published^74^ with salient points described here. We followed the procedures described in the Cochrane Handbook for Systematic Reviews of Interventions^75^.

### Deviation from published protocol

We planned to use Grading of Recommendations Assessment Development and Evaluation (GRADE^76^) approach to rate the quality of evidence for primary outcomes and the important secondary outcomes; however, there was substantial missing information in the papers, so we were unable to apply the GRADE approach (see Supplementary Results 2 for our limited GRADE exercise).

### Search strategy

Table 4 gives details of the search strategy developed to detect randomised controlled trials (RCTs) and controlled clinical trials of ‘Pulmonary Rehabilitation’ AND ‘COPD or other CRD’ AND ‘LMIC or low-resource settings’ from 1990 (when global COPD guidelines first recommended PR^77^) to November 2018 with no language restrictions. We searched MEDLINE (Supplementary Methods 1) EMBASE, Global Health (CABI), AMED, PubMed, and the Cochrane Database of Controlled Trials (CENTRAL). We did not undertake hand searching as we found no journal that regularly published PR papers in LMICs. Additionally, we conducted forward citations of the included articles. We used EndNote for overall data management.

**Table 4:** PICOS search strategy.

PICOS	Description, inclusion/exclusion criteria	Operational rules
Population	Adults with CRDs. Comorbidity was not an exclusion criterion. No age restrictions	Any CRD (COPD, post TB, remodelled asthma, bronchiectasis, interstitial lung disease) or poorly differentiated respiratory conditions that cause chronic symptoms. We excluded studies that included non-respiratory causes for symptoms
Intervention	Pulmonary rehabilitation (PR), which comprised both exercise AND at least one non-exercise component	Non-exercise components included recognised PR interventions, such as patient education, breathing exercises, energy conservation training, self-management skill development We included optimisation of pharmacotherapy as a component because in low-resource settings this may not be accessed/provided elsewhere
Comparison	Population who are not given PR	Individuals received usual care as normal in the setting
Outcomes	Primary outcomes: • Functional exercise capacity • Health-related quality of life (HRQoL) Secondary outcomes: • Symptom control • Psychological status • Uptake of the service, completion rates • Adverse effects	Validated instruments considered: *Functional exercise capacity*: 6-Minute Walk Test, Endurance Shuttle Walking Test *HRQoL:* SGRQ, CRQ, SF-36, SF-12, EQ-5D *Symptom control*: mMRC, Borg scale *Psychological status*: HADS, PHQ-9, STAI, Beck Inventory test Non-validated instruments were extracted, but evidence noted as being less reliable
Setting	Low-resource settings Typically characterised by a lack of funds leading to: • Limited access to medication, equipment • Poorly developed infrastructure • Few trained personnel • Limited access to routine care	In practice, this decision was normally based on the World Bank category of a LMIC country at the time of the study. However, while low resource settings were usually in LMICs, PR delivered in a well-resourced context (e.g. a tertiary care hospital) in an LMIC would be excluded, and interventions in HICs might be included if the context was low resource (e.g. remote, deprived community)
Study designs	Randomised controlled trials (RCTs); clinical controlled trials	We excluded studies that did not have a control group

*SGRQ* St Georges Respiratory Questionnaire, *CRQ* Chronic Respiratory Questionnaire, *SF-36* Short Form-36, *SF-12* Short Form-12, *EQ-5D* EuroQol Five Dimension, *mMRC* modified Medical Research Council, *HADS* Hospital Anxiety and Depression Scale, *PHQ-9* Patient Health Questionnaire-9, *STAI* State-Trait Anxiety Inventory.

The searches were completed on 28 October 2018, with a pre-publication update on 8 March 2020 using the ‘efficient and effective’ approach^78^ of forward citation using Google Scholar, of all included papers, and the Cochrane review^23^.

### Selection process

Details of inclusion and exclusion criteria and definitions used are in Table 4. In summary, we undertook a duplicate selection process using rules for operationalising the inclusion/exclusion criteria (see protocol for details^74^). Two trained reviewers (G.M.M.H. and M.N.U.) independently screened titles and abstracts, then full-text papers (G.M.M.H., M.N.U., and K.D.). Disagreements were resolved by discussion, involving H.P. and R.R. or the wider team as necessary. We reported the process in a PRISMA flow diagram (Fig. 1)^79^.

### Outcome measurement

Our primary outcomes were between-group difference in functional exercise capacity (e.g. 6-MWT^80–82^) and HRQoL (e.g. SGRQ^83,84^). We also included breathlessness (e.g. mMRC Dyspnoea score^85^). These are defined, and secondary outcomes are described in Table 4.

### Data extraction and RoB

Two reviewers (G.M.M.H. and M.N.U. and checked by H.P.) extracted data on a piloted data extraction form (Supplementary Methods 2) based on the Cochrane Effective Practice and Organisation of Care guidance^86^; G.M.M.H. and M.N.U. (checked by H.P.) independently assessed the methodological quality of all the included studies according to the Cochrane RoB tool^75^.

### Data analysis

The analysis addressed our three objectives:

1. *Effectiveness of PR in low-resource settings*: On the basis of our initial scoping, we anticipated that our included studies would have substantial clinical, methodological, and statistical heterogeneity, and meta-analysis would not be appropriate. We, therefore, conducted a narrative synthesis illustrating the key outcomes on a harvest plot^87,88^. In order to ensure transparency of interpretation, the decisions that underpinned the harvest plot are described in Table 1: column 5.
2. *Components used in effective studies*: We identified the components that are described in internationally recognised guidelines^13,15,89^ using categories from the American Thoracic Society/European Respiratory Society task force report^13^, British Thoracic Society guidelines for PR^15^, and Lung Foundation of Australia^90^. We then constructed a matrix with the components used in the (effective and ineffective) studies.
3. *Models of care used in the PR interventions*: We described the models of care used, including PR providers and (if specified) their training, venue and equipment available, number and frequency of training sessions, use of telehealth, and strategies for sustainability.

## Supplementary information

Supplementary Information

## Data Availability

Data sharing is not applicable as no data sets were produced during this study. The data that support the findings of this systematic review are all available in the published papers.

## References

[1] Ahmed R, Robinson R, Mortimer K (2017). The epidemiology of noncommunicable respiratory disease in sub-Saharan Africa, the Middle East, and North Africa. Malawi Med. J..

[2] Shayo FK, Bintabara D (2019). Are Tanzanian health facilities ready to provide management of chronic respiratory diseases? An analysis of national survey for policy implications. PLoS ONE.

[3] World Health Organisation. Global alliance against chronic respiratory diseases. Global surveillance, prevention and control of chronic respiratory diseases. A comprehensive approach. https://www.who.int/respiratory/en/ (2007).

[4] Ferkol T, Schraufnagel D (2014). The global burden of respiratory disease. Ann. Am. Thorac. Soc..

[5] India State-Level Disease Burden Initiative CRD Collaborators. The burden of chronic respiratory diseases and their heterogeneity across the states of India: the Global Burden of Disease Study 1990-2016. *Lancet Glob. Health* **6**, e1363–e1374 (2018). 10.1016/S2214-109X(18)30409-1 PMC6227385 30219316

[6] GBD 2017 DALYs and HALE Collaborators. Global, regional, and national disability-adjusted life-years (DALYs) for 359 diseases and injuries and healthy life expectancy (HALE) for 195 countries and territories, 1990-2017: a systematic analysis for the Global Burden of Disease Study 2017. *Lancet* **392**, 1859–1922 (2018). 10.1016/S0140-6736(18)32335-3 PMC6252083 30415748

[7] Global Burden of Disease Study 2013 Collaborators. Global, regional, and national incidence, prevalence, and years lived with disability for 301 acute and chronic diseases and injuries in 188 countries, 1990-2013: a systematic analysis for the Global Burden of Disease Study 2013. *Lancet* **386**, 743–800 (2015). 10.1016/S0140-6736(15)60692-4 PMC4561509 26063472

[8] Adeloye D (2015). Global and regional estimates of COPD prevalence: systematic review and meta-analysis. J. Glob. Health.

[9] Alam DS, Chowdhury MA, Siddiquee AT, Ahmed S, Clemens JD (2015). Prevalence and determinants of chronic obstructive pulmonary disease (COPD) in Bangladesh. COPD.

[10] Islam MS, Hossain MM, Pasha MM, Azad AK, Murshed KM (2013). Prevalence and risk factors of chronic obstructive pulmonary disease (COPD) in Dhaka city population. Mymensingh Med. J..

[11] Ait-Khaled N, Enarson D, Bousquet J (2001). Chronic respiratory diseases in developing countries: the burden and strategies for prevention and management. Bull. World Health Organ..

[12] Troosters T, Blondeel A, Rodrigues FM, Janssens W, Demeyer H (2019). Strategies to increase physical activity in chronic respiratory diseases. Clin. Chest Med..

[13] Rochester CL (2015). An Official American Thoracic Society/European Respiratory Society Policy Statement: enhancing implementation, use, and delivery of pulmonary rehabilitation. Am. J. Respir. Crit. Care Med..

[14] Global Initiative for Chronic Obstractive Lung Disease. Pocket guide to COPD diagnosis, management and prevention. https://goldcopd.org/ (2019).

[15] Bolton CE (2013). British Thoracic Society guideline on pulmonary rehabilitation in adults. Thorax.

[16] Golmohammadi K, Jacobs P, Sin DD (2004). Economic evaluation of a community-based pulmonary rehabilitation program for chronic obstructive pulmonary disease. Lung.

[17] Griffiths TL, Phillips CJ, Davies S, Burr ML, Campbell IA (2001). Cost effectiveness of an outpatient multidisciplinary pulmonary rehabilitation programme. Thorax.

[18] Puhan, M., Scharplatz, M., Troosters, T., Walters, E. H. & Steurer, J. Pulmonary rehabilitation following exacerbations of chronic obstructive pulmonary disease. *Cochrane Database Syst. Rev*. Cd005305 (2009). 10.1002/14651858.CD005305.pub2 19160250

[19] Puhan MA, Gimeno-Santos E, Cates CJ, Troosters T (2016). Pulmonary rehabilitation following exacerbations of chronic obstructive pulmonary disease. Cochrane Database Syst. Rev..

[20] Seymour JM (2010). Outpatient pulmonary rehabilitation following acute exacerbations of COPD. Thorax.

[21] Araujo ZTS (2019). Pulmonary rehabilitation for people with chronic obstructive pulmonary disease: A protocol for an overview of Cochrane reviews. Medicine.

[22] Jenkins AR (2018). Efficacy of supervised maintenance exercise following pulmonary rehabilitation on health care use: a systematic review and meta-analysis. Int. J. Chron. Obstruct. Pulmon. Dis..

[23] McCarthy, B. et al. Pulmonary rehabilitation for chronic obstructive pulmonary disease. *Cochrane Database Syst. Rev*. Cd003793 (2015). 10.1002/14651858.CD003793.pub3 PMC10008021 25705944

[24] Roberts NJ, Kidd L, Kirkwood K, Cross J, Partridge MR (2018). A systematic review of the content and delivery of education in pulmonary rehabilitation programmes. Respir. Med..

[25] Black RE (1990). Prevention in developing countries. J. Gen. Intern. Med..

[26] Gothi D, Joshi JM (2011). Pulmonary rehabilitation in resource poor settings. Indian J. Chest Dis. Allied Sci..

[27] Han W (2012). Health care system reforms in developing countries. J. Public Health Res..

[28] Kumar R (2019). Universal health coverage - time to dismantle vertical public health programs in India. J. Fam. Med. Prim. Care.

[29] Heine M (2019). Exercise-based rehabilitation for major non-communicable diseases in low-resource settings: a scoping review. BMJ Glob. Health.

[30] Forum of International Respiratory Societies. The global impact of respiratory disease. https://www.who.int/gard/publications/The_Global_Impact_of_Respiratory_Disease.pdf (2017).

[31] Brooks D, Lacasse Y, Goldstein RS (1999). Pulmonary rehabilitation programs in Canada: national survey. Can. Respir. J..

[32] Wadell K (2013). Hospital-based pulmonary rehabilitation in patients with COPD in Sweden-a national survey. Respir. Med..

[33] Yohannes AM, Connolly MJ (2004). Pulmonary rehabilitation programmes in the UK: a national representative survey. Clin. Rehabil..

[34] Desalu OO (2013). Guideline-based COPD management in a resource-limited setting - physicians’ understanding, adherence and barriers: a cross-sectional survey of internal and family medicine hospital-based physicians in Nigeria. Prim. Care Respir. J..

[35] Akinci AC, Olgun N (2011). The effectiveness of nurse-led, home-based pulmonary rehabilitation in patients with COPD in Turkey. Rehabil. Nurs..

[36] de Grass D, Manie S, Amosum S (2014). Effectiveness of a home-based pulmonary rehabilitation programme in pulmonary function and health related quality of life for patients with pulmonary tuberculosis: a pilot study. Afr. Health Sci..

[37] de Souto Araujo ZT (2012). Effectiveness of low-intensity aquatic exercise on COPD: a randomized clinical trial. Respir. Med..

[38] Deepak TH, Mohapatra PR, Janmeja AK, Sood P, Gupta M (2014). Outcome of pulmonary rehabilitation in patients after acute exacerbation of chronic obstructive pulmonary disease. Indian J. Chest Dis. Allied Sci..

[39] Duruturk N, Arıkan H, Ulubay G, Tekindal MA (2016). A comparison of calisthenic and cycle exercise training in chronic obstructive pulmonary disease patients: a randomized controlled trial. Expert Rev. Respir. Med..

[40] Elci A, Börekçi Ş, Ovayolu N, Elbek O (2008). The efficacy and applicability of a pulmonary rehabilitation programme for patients with COPD in a secondary-care community hospital. Respirology.

[41] Farias CC (2014). Costs and benefits of pulmonary rehabilitation in chronic obstructive pulmonary disease: a randomized controlled trial. Braz. J. Phys. Ther..

[42] Ghanem M, ELaal EA, Mehany M, Tolba K (2010). Home-based pulmonary rehabilitation program: effect on exercise tolerance and quality of life in chronic obstructive pulmonary disease patients. Ann. Thorac. Med..

[43] Karapolat H (2007). Do the benefits gained using a short-term pulmonary rehabilitation program remain in COPD patients after participation?. Lung.

[44] Mohammadi F, Jowkar Z, Reza Khankeh H, Fallah Tafti S (2013). Effect of home-based nursing pulmonary rehabilitation on patients with chronic obstructive pulmonary disease: a randomised clinical trial. Br. J. Community Nurs..

[45] Paz-Díaz H, De Oca MM, López JM, Celli BR (2007). Pulmonary rehabilitation improves depression, anxiety, dyspnea and health status in patients with COPD. Am. J. Phys. Med. Rehabil..

[46] Pradella CO (2015). Home-based pulmonary rehabilitation for subjects with COPD: a randomized study. Respir. Care.

[47] Singh V, Khandelwal DC, Khandelwal R, Abusaria S (2003). Pulmonary rehabilitation in patients with chronic obstructive pulmonary disease. Indian J. Chest Dis. Allied Sci..

[48] Bennett S (2012). Influencing policy change: the experience of health think tanks in low-and middle-income countries. Health Policy Plan..

[49] Grover S (2017). Clinical trials in low and middle-income countries—successes and challenges. Gynecol. Oncol. Rep..

[50] Osei A (2012). Challenges of clinical trials in low-and middle-income countries. Int. Psychiatry.

[51] Maters GA, Sanderman R, Kim AY, Coyne JC (2013). Problems in cross-cultural use of the hospital anxiety and depression scale: “no butterflies in the desert”. PLoS ONE.

[52] Singh SJ, Halpin DM, Salvi S, Kirenga BJ, Mortimer K (2019). Exercise and pulmonary rehabilitation for people with chronic lung disease in LMICs: challenges and opportunities. Lancet Respir. Med..

[53] Sheikh, A. et al. RESPIRE: The National Institute for Health Research’s (NIHR) Global Respiratory Health Unit. *J. Glob. Health* **8**, 020101 (2018). 10.7189/jogh.08.020101 PMC6304165 30603074

[54] Free, R. C. et al. The RECHARGE database: towards a global standard for pulmonary rehabilitation (PR). *Eur. Respir. J*. **54**, PA693 (2019).

[55] Wiysonge, C. S. et al. Financial arrangements for health systems in low-income countries: an overview of systematic reviews. *Cochrane Database Syst. Rev*. CD011084 (2017). 10.1002/14651858.CD011084.pub2 PMC5618470 28891235

[56] Khan JAM, Ahmed S, Evans TG (2017). Catastrophic healthcare expenditure and poverty related to out-of-pocket payments for healthcare in Bangladesh-an estimation of financial risk protection of universal health coverage. Health Policy Plan..

[57] Mortimer K, Cuevas L, Squire B, Thomson R, Tolhurst R (2015). Improving access to effective care for people with chronic respiratory symptoms in low and middle income countries. BMC Proc..

[58] Troosters T, Gosselink R, Janssens W, Decramer M (2010). Exercise training and pulmonary rehabilitation: new insights and remaining challenges. Eur. Respir. Rev..

[59] Pfeifer K, Geidl W (2017). Physical activity recommendations for adults with a chronic disease: methods, database and rationale. Gesundheitswesen.

[60] Quaak M, Schayck Van, Knaapen C, Van A, Schooten F (2009). Genetic variation as a predictor of smoking cessation success. A promising preventive and intervention tool for chronic respiratory diseases?. Eur. Respir. J..

[61] Rajendra K, Shukla SD, Gautam SS, Hansbro PM, O’Toole RF (2018). The role of environmental exposure to non-cigarette smoke in lung disease. Clin. Transl. Med..

[62] Varkey AB (2004). Chronic obstructive pulmonary disease in women: exploring gender differences. Curr. Opin. Pulm. Med..

[63] Furlan AD (2018). Rehabilitation service models for people with physical and/or mental disability living in low-and middle-income countries: a systematic review. J. Rehabil. Med..

[64] Heine M (2019). Patient-centred rehabilitation for non-communicable disease in a low-resource setting: study protocol for a feasibility and proof-of-concept randomised clinical trial. BMJ Open.

[65] Horton EJ (2018). Comparison of a structured home-based rehabilitation programme with conventional supervised pulmonary rehabilitation: a randomised non-inferiority trial. Thorax.

[66] José A (2017). Does home-based pulmonary rehabilitation improve functional capacity, peripheral muscle strength and quality of life in patients with bronchiectasis compared to standard care?. Braz. J. Phys. Ther..

[67] Liu XL (2014). Effectiveness of home-based pulmonary rehabilitation for patients with chronic obstructive pulmonary disease: a meta-analysis of randomized controlled trials. Rehabil. Nurs..

[68] Macrea, M., ZuWallack, R. & Nici, L. There’s no place like home: integrating pulmonary rehabilitation into the home setting. *Monaldi Arch. Chest Dis*. **87**, 859 (2017). 10.4081/monaldi.2017.859 28967733

[69] Zwerink M (2016). Cost-effectiveness of a community-based exercise programme in COPD self-management. COPD.

[70] Pinnock H, McKinstry B (2016). Digital technology in respiratory diseases: promises,(no) panacea and time for a new paradigm. Chron. Respir. Dis..

[71] Hutchinson A, Barclay-Klingle N, Galvin K, Johnson MJ (2018). Living with breathlessness: a systematic literature review and qualitative synthesis. Eur. Respir. J..

[72] Siddharthan T (2018). Effectiveness-implementation of COPD case finding and self-management action plans in low-and middle-income countries: global excellence in COPD outcomes (GECo) study protocol. Trials.

[73] Patel MR (2018). Improving the affordability of prescription medications for people with chronic respiratory disease. An official American Thoracic Society policy statement. Am. J. Respir. Crit. Care Med..

[74] Habib GMM (2019). Systematic review (protocol) of clinical effectiveness and models of care of low-resource pulmonary rehabilitation. NPJ Prim. Care Respir. Med..

[75] Higgins, J. P. T. & Green, S. (eds) *Cochrane Handbook for Systematic Reviews of Interventions Version 5.1.0 [updated March 2011]* (The Cochrane Collaboration, 2011).

[76] GRADE Working Group. Grading of recommendations assessment, development and evaluation (GRADE). https://www.gradeworkinggroup.org/ (2012).

[77] Casaburi R (2008). A brief history of pulmonary rehabilitation. Respir. Care.

[78] Greenhalgh T, Peacock R (2005). Effectiveness and efficiency of search methods in systematic reviews of complex evidence: audit of primary sources. BMJ.

[79] Liberati A (2009). The PRISMA statement for reporting systematic reviews and meta-analyses of studies that evaluate healthcare interventions: explanation and elaboration. Ann. Int. Med..

[80] Bohannon RW, Crouch R (2017). Minimal clinically important difference for change in 6-minute walk test distance of adults with pathology: a systematic review. J. Eval. Clin. Pract..

[81] Lima CA (2018). Six-minute walk test as a determinant of the functional capacity of children and adolescents with cystic fibrosis: a systematic review. Respir. Med..

[82] Brooks GC (2015). Accuracy and usability of a self-administered 6-minute walk test smartphone application. Circ. Heart Fail..

[83] American Thoracic Society (ATS). St. George’s Respiratory Questionnaire (SGRQ). https://www.thoracic.org/members/assemblies/assemblies/srn/questionaires/sgrq.php (2016).

[84] Jones P, Quirk F, Baveystock C (1991). The St George’s Respiratory Questionnaire. Respir. Med..

[85] Munari AB (2018). Modified Medical Research Council dyspnea scale in GOLD classification better reflects physical activities of daily living. Respir. Care.

[86] Effective Practice and Organisation of Care (EPOC) Group. Data collection form. EPOC resources for review authors. https://epoc.cochrane.org/about-us (2013).

[87] Ogilvie D (2008). The harvest plot: a method for synthesising evidence about the differential effects of interventions. BMC Med. Res. Methodol..

[88] Crowther M, Avenell A, MacLennan G, Mowatt G (2011). A further use for the harvest plot: a novel method for the presentation of data synthesis. Res. Synth. Methods.

[89] Spruit MA (2013). An official American Thoracic Society/European Respiratory Society statement: key concepts and advances in pulmonary rehabilitation. Am. J. Respir. Crit. Care Med..

[90] Alison JA (2017). Australian and New Zealand Pulmonary Rehabilitation Guidelines. Respirology.

