# Supplementary material for: Systematic review of clinical effectiveness, components, and delivery of pulmonary rehabilitation in low-resource settings
Source: NPJ Prim Care Respir Med. 2020 Nov 19;30:52. doi: 10.1038/s41533-020-00210-y (PMC7677536; doi:10.1038/s41533-020-00210-y)
Supplement: Supplementary file 1 — Supplementary Information [file 41533_2020_210_MOESM1_ESM.pdf]

**Supplementary methods 1. Medline search strategy**

| SI | Searches of MEDLINE on 28 Oct. 18 | Results |
|---|---|---|
| 1 | exp Lung Diseases, Obstructive/ | 197888 |
| 2 | pulmonary disease, chronic obstructive.mp. or exp Pulmonary Disease, Chronic Obstructive/ | 49651 |
| 3 | emphysema$.mp. | 31795 |
| 4 | (Chronic$ adj3 bronchiti$).mp. | 10714 |
| 5 | (obstruct$ adj3 (pulmonary or lung$ or airway$ or airflow$ or bronch$ or respirat$)).mp. | 98543 |
| 6 | COPD.mp. | 34381 |
| 7 | COAD.mp. | 222 |
| 8 | COBD.mp. | 12 |
| 9 | AECB.mp. | 210 |
| 10 | (obstruct* adj3 (pulmonary or lung* or airway* or airflow* or bronch* or respirat*)).mp. | 98543 |
| 11 | AECOPD.mp. | 630 |
| 12 | exp chronic bronchitis/ | 1688 |
| 13 | 1 or 2 or 3 or 4 or 5 or 6 or 7 or 8 or 9 or 10 or 11 or 12 | 252449 |
| 14 | REHABILITATION.mp. or exp REHABILITATION/ | 459193 |
| 15 | Respiratory Therapy.mp. or exp Respiratory Therapy/ | 106770 |
| 16 | Physical Therapy Modalities.mp. or exp Physical Therapy Modalities/ | 138907 |
| 17 | (rehabilitat* or fitness* or exercis* or train* or physiotherap* or (physical* adj therap*)).mp. | 968222 |
| 18 | 14 or 15 or 16 or 17 | 1197761 |
| 19 | (Africa or Asia or Caribbean or "West Indies" or "South America" or "Latin America" or "Central America").mp. | 220128 |
| 20 | (Afghanistan or Albania or Algeria or Angola or Antigua or Barbuda or Argentina or Armenia or Armenian or Aruba or Azerbaijan or Bahrain or Bangladesh or Barbados or Benin or Byelarus or Byelorussian or Belarus or Belorussian or Belorussia or Belize or Bhutan or Bolivia or Bosnia or Herzegovina or Hercegovina or Botswana or Brasil or Brazil or Bulgaria or "Burkina Faso" or "Burkina Fasso" or "Upper Volta" or Burundi or Urundi or Cambodia or "Khmer Republic" or Kampuchea or Cameroon or Cameroons or Cameron or Camerons or "Cape Verde" or "Central African Republic" or Chad or Chile or China or Colombia or Comoros or "Comoro Islands" or Comores or Mayotte or Congo or Zaire or "Costa Rica" or "Cote d'Ivoire" or "Ivory Coast" or Croatia or Cuba or Cyprus or Czechoslovakia or "Czech Republic" or Slovakia or "Slovak Republic").mp. | 436693 |
| 21 | (Djibouti or "French Somaliland" or Dominica or "Dominican Republic" or "East Timor" or "East Timur" or "Timor Leste" or Ecuador or Egypt or "United Arab Republic" or "El Salvador" or Eritrea or Estonia or Ethiopia or Fiji or Gabon or "Gabonese Republic" or Gambia or Gaza or Georgia or Georgian or Ghana or "Gold Coast" or Greece or Grenada or Guatemala or Guinea or Guam or Guiana or Guyana or Haiti or Honduras or Hungary or India or Maldives or Indonesia or Iran or Iraq or "Isle of Man" or Jamaica or | 516935 |

| | | |
|---|---|---|
| | Jordan or Kazakhstan or Kazakh or Kenya or Kiribati or Korea or Kosovo or Kyrgyzstan or Kirghizia or "Kyrgyz Republic" or Kirghiz or Kirgizstan or "Lao PDR" or Laos or Latvia or Lebanon or Lesotho or Basutoland or Liberia or Libya or Lithuania).mp. | |
| 22 | (Macedonia or Madagascar or "Malagasy Republic" or Malaysia or Malaya or Malay or Sabah or Sarawak or Malawi or Nyasaland or Mali or Malta or "Marshall Islands" or Mauritania or Mauritius or "Agalega Islands" or Mexico or Micronesia or "Middle East" or Moldova or Moldovia or Moldovian or Mongolia or Montenegro or Morocco or Ifni or Mozambique or Myanmar or Myanma or Burma or Namibia or Nepal or "Netherlands Antilles" or "New Caledonia" or Nicaragua or Niger or Nigeria or "Northern Mariana Islands" or Oman or Muscat or Pakistan or Palau or Palestine or Panama or Paraguay or Peru or Philippines or Philipines or Phillipines or Phillippines or Poland or Portugal or "Puerto Rico").mp. | 274812 |
| 23 | (Romania or Rumania or Roumania or Russia or Russian or Rwanda or Ruanda or "Saint Kitts" or "St Kitts" or Nevis or "Saint Lucia" or "St Lucia" or "Saint Vincent" or "St Vincent" or Grenadines or Samoa or "Samoan Islands" or "Navigator Island" or "Navigator Islands" or "Sao Tome" or "Saudi Arabia" or Senegal or Serbia or Montenegro or Seychelles or "Sierra Leone" or Slovenia or "Sri Lanka" or Ceylon or "Solomon Islands" or Somalia or Sudan or Suriname or Surinam or Swaziland or Syria or Tajikistan or Tadzhikistan or Tadjikistan or Tadzhik or Tanzania or Thailand or Togo or "Togolese Republic" or Tonga or Trinidad or Tobago or Tunisia or Turkey or Turkmenistan or Turkmen or Uganda or Ukraine or Uruguay or USSR or "Soviet Union" or "Union of Soviet Socialist Republics" or Uzbekistan or Uzbek or Vanuatu or "New Hebrides" or Venezuela or Vietnam or "Viet Nam" or "West Bank" or Yemen or Yugoslavia or Zambia or Zimbabwe or Rhodesia).mp. | 328981 |
| 24 | ((developing or "less* developed" or "under developed" or underdeveloped or "middle income" or "low* income" or underserved or "under served" or deprived or poor*) adj (countr* or nation* or population* or world)).mp. | 127486 |
| 25 | ((developing or "less* developed" or "under developed" or underdeveloped or "middle income" or "low* income") adj (economy or economies)).mp. | 351 |
| 26 | (low* adj (GDP or GNP or "gross domestic" or "gross national")).mp. | 203 |
| 27 | (low adj3 middle adj3 countr*).mp. | 8403 |
| 28 | (LMIC or LMICs or "third world" or "LAMI country" or "LAMI countries").mp. | 4711 |
| 29 | ("transitional country" or "transitional countries").mp. | 131 |
| 30 | (setting* adj2 (resource* or poor* or constrain* or low*)).mp. | 12989 |
| 31 | 19 or 20 or 21 or 22 or 23 or 24 or 25 or 26 or 27 or 28 or 29 or 30 | 1629217 |
| 32 | 13 and 18 and 31 | 1370 |

**Supplementary methods 2. Piloted data extraction form**

Notes on using this data extraction form:

- Be consistent in the order and style used to describe the information for each included study
- Record any missing information as unclear or not described, to make it clear that the information was not found in the study report
- Include any instructions and decision rules on the data collection form, or in an accompanying document.

| | |
|---|---|
| Study ID *(surname of first author and year first full report of study was published)* | |
| Notes: | |

### 1. General information

| | |
|---|---|
| Date of data extraction *(dd/mm/yyyy)* | |
| Name of reviewer | |
| Article title | |
| Reference *(journal, year, volume, issue, pages)* | |
| Country of origin | |
| Type of publication *(e.g. full report, conference abstract)* | |
| Notes: | |

### 2. Specific information

| | |
|---|---|
| Aim of study (*process & clinical objectives)* | |
| Study design | ☐ RCT ☐ CCT |
| Start date | |
| End date | |
| Duration of participation | |
| Number of centre(s) in study | |
| Notes: | |

| Population and setting | |
|---|---|
| Health condition targeted | ☐COPD ☐Post TB ☐Remodelled asthma ☐ Bronchiectasis ☐ ILD ☐ Undifferentiated CRD |
| Source/ setting of the population *(e.g. urban, rural, particular ethnic group)* | |
| Setting | |
| Notes: | |

| Patients | |
|---|---|
| Inclusion criteria | |
| Exclusion criteria | |
| Recruitment procedures used | |
| Number recruited | |
| Number randomised | |
| Number completed | |
| Age | |
| Sex | |
| Other relevant characteristics *(please specify)* | |
| Notes: | |

**Intervention & Control**

| Level 1: Components of PR | |
|---|---|
| Intervention summary | the Department of Pulmonary Medicine supervised the activities. |
| Components of PR | ☐ xxxxx<br>☐ xxxxx<br>☐ xxxxx<br>☐ xxxxx<br>☐ other |
| **Level2: Models of service delivery** | |
| Brief name | |
| Why: rationale/theory/goal | |
| What: physical/ informational materials used | |
| What: procedures/activities/ processes used | |

| | |
|---|---|
| Who: provider(s), expertise, background, training | |
| How: modes of delivery | ☐ face-to-face ☐ telephone ☐ internet ☐ video/DVD ☐ written materials ☐ other *(specify)*<br><br>individual ☐ group ☐ |
| Where: type(s) of location(s) where intervention occurred | |
| When and How Much | number of sessions:<br>session schedule:<br>session duration: |
| Tailoring *(if applicable: what, why, when, and how)* | |
| Modifications *(if applicable: what, why, when, and how)* | |
| How Well: intervention adherence/ fidelity assessment methods | |
| How Well: intervention adherence/ fidelity assessment results | |
| Notes: | |
| **Level 3: Implementation strategies** | |
| Type of intervention *(EPOC TAXONOMY)* | ☐ audit & feedback<br>☐ clinical incident reporting<br>☐ monitoring the performance of the delivery of healthcare<br>☐ communities of practice<br>☐ continuous quality improvement<br>☐ educational games<br>☐ educational materials<br>☐educational meetings<br>☐ educational outreach visits/academic detailing<br>☐ clinical practice guidelines<br>☐ inter-professional education<br>☐ local consensus processes<br>☐ local opinion leaders<br>☐ managerial supervision<br>☐ patient-mediated interventions<br>☐ public release of performance data<br>☐ reminders |

| | |
|---|---|
| | ☐ routine patient-reported outcome measures<br>☐ tailored interventions |

| **Outcomes** | |
|---|---|
| **Primary** | |
| Functional exercise capacity | ☐ |
| Health-Related Quality of Life (HRQoL) | ☐ |
| **Secondary** | |
| Symptom control | ☐ |
| Psychological status | ☐ |
| Health care burden | ☐ |
| Uptake of the service, completion rates | ☐ |
| Adverse effects | ☐ |

| Full primary outcome description | Method(s) of measurement | Time points measured *(e.g. 6 months)* | Time points reported *(e.g. 6 months)* |
|---|---|---|---|
| Functional exercise capacity | | | |
| HRQoL | | | |
| Notes: | | | |

| Full secondary outcome description | Method(s) of measurement | Time points measured *(e.g. 6 months)* | Time points reported *(e.g. 6 months)* |
|---|---|---|---|
| Symptom control | | | |
| Notes: | | | |

| **Analysis** | |
|---|---|
| Sample size/power calculation | |
| Method(s) of analysis *(statistical analysis and approach, e.g. ITT/per protocol)* | |
| Missing data and how addressed | |
| Drop-out rate | |
| Notes: | |

**Results**

| |
|---|
| [Complete Excel Doc] |
| Notes: |

| |
|---|
| |

## 3. Other information

| | |
|---|---|
| Limitations | |
| Key conclusions of study authors | |
| Generalisability | |
| Potentially relevant references | |
| Funding | |
| Ethical approval | |
| Corresponding author contact details | |
| Other | |
| Notes: | |

## 4. Risk of bias assessment

*Source: Higgins JPT, Green S (editors). Cochrane Handbook for Systematic Reviews of Interventions. Version 5.1.0 [updated March 2011]. The Cochrane Collaboration, 2011. Available at: [www.cochrane-handbook.org](www.cochrane-handbook.org)*
*See guidance notes.*

| **Domain** | **Risk of bias** | | | | **Support for judgment** |
|---|---|---|---|---|---|
| | **Low** | **High** | **Unclear** | **Not applicable** | |
| Random sequence generation *(selection bias)* | ☐ | ☐ | ☐ | ☐ | |
| Allocation concealment *(selection bias)* | ☐ | ☐ | ☐ | ☐ | |
| Blinding of participants and personnel *(performance bias)* | ☐ | ☐ | ☐ | ☐ | |
| Blinding of outcome assessment *(detection bias)* | ☐ | ☐ | ☐ | ☐ | |
| Incomplete outcome data *(attrition bias)* | ☐ | ☐ | ☐ | ☐ | |
| Selective outcome reporting *(reporting bias)* | ☐ | ☐ | ☐ | ☐ | |
| Other bias | ☐ | ☐ | ☐ | ☐ | |
| Overall risk of bias | ☐ | ☐ | ☐ | ☐ | |
| Notes: | | | | | |

**Supplementary results 1. Risk of Bias assessment**

| Article | Random | Conceale d | Personne l blinded | Outcome s blinded | Attrition | Selective reporting | Other bias | Overall |
|---|---|---|---|---|---|---|---|---|
| Akinci 2011 | ☹ | ☹ | ☹ | 😐 | ☹ | 😐 | ☹ | ☹ |
| de Grass 2014 | ☺ | ☺ | ☹ | ☺ | ☹ | 😐 | ☺ | 😐 |
| Deepak 2014 | ☺ | 😐 | ☹ | 😐 | 😐 | 😐 | 😐 | ☹ |
| De Souto Araujo 2012 | ☺ | 😐 | ☹ | 😐 | ☹ | 😐 | ☹ | ☹ |
| Duruturk 2015 | ☺ | ☺ | ☹ | 😐 | ☺ | 😐 | ☺ | 😐 |
| Elci 2008 | ☺ | 😐 | ☹ | ☹ | 😐 | 😐 | 😐 | ☹ |
| Farias 2014 | ☺ | 😐 | ☹ | 😐 | ☺ | 😐 | ☹ | ☹ |
| Ghanem 2010 | ☺ | 😐 | ☹ | ☹ | 😐 | 😐 | ☺ | ☹ |
| Karapolat 2007 | ☺ | 😐 | ☹ | ☹ | ☹ | 😐 | 😐 | ☹ |
| Mohammadi 2013 | ☺ | 😐 | ☹ | ☹ | 😐 | 😐 | 😐 | ☹ |
| Paz-Diaz 2007 | ☺ | 😐 | ☹ | 😐 | 😐 | 😐 | 😐 | ☹ |
| Pradella 2015 | ☺ | 😐 | ☹ | 😐 | ☺ | 😐 | ☹ | ☹ |
| Singh 2003 | ☺ | 😐 | ☹ | 😐 | 😐 | 😐 | 😐 | ☹ |

☹ High risk

😐 Moderate risk

☺ Low risk

**Supplementary results 2. GRADE assessment**

| **Clinical effectiveness and models of care of Low-Resource Pulmonary Rehabilitation** | | | | | | |
|---|---|---|---|---|---|---|
| **Population:** Adults with Chronic Respiratory Diseases including undiagnosed condition that cause chronic respiratory symptoms<br>**Intervention:** Pulmonary Rehabilitation<br>**Settings:** Low-resource settings<br>**Comparison:** Usual care | | | | | | |
| **Outcomes** | **Illustrative comparative risks* (95% CI)** | | **Relative effect (95% CI)** | **No of Participants (studies involved)** | **Quality of the evidence (GRADE)** | **Comments** |
| | **Assumed risk** | **Corresponding risk** | | | | |
| | **Usual care** | **Pulmonary rehabilitation** | | | | |
| **Functional exercise capacity** | See comment | See comment | Not estimable | 509 (11) | See comment | The effect is uncertain. Nine of the studies had high risk of bias and two had moderate risk of bias. All the studies did not have same scale of outcome measurement. Four studies measured effectiveness by between group comparison whereas seven studies measured within group comparison. |
| **Health-Related Quality of Life** | See comment | See comment | Not estimable | 506(12) | See comment | The effect is uncertain. Eleven of the studies had high risk of bias and one had moderate |

| | | | | | | |
|---|---|---|---|---|---|---|
| | | | | | | risk of bias. All the studies did not have same scale of outcome measurement. Five studies measured effectiveness by between group comparison whereas seven studies measured within group comparison. |
| **Breathlessness** | See comment | See comment | Not estimable | 455(11) | See comment | The effect is uncertain. Nine of the studies had high risk of bias and two had moderate risk of bias. All the studies did not have same scale of outcome measurement. Four studies measured effectiveness by between group comparison whereas seven studies measured within group comparison. |
